# Prepulse inhibition in *Drosophila melanogaster* larvae

**DOI:** 10.1242/bio.034710

**Published:** 2018-09-15

**Authors:** Yutaro Matsumoto, Kazuya Shimizu, Kota Arahata, Miku Suzuki, Akira Shimizu, Koki Takei, Junji Yamauchi, Satoko Hakeda-Suzuki, Takashi Suzuki, Takako Morimoto

**Affiliations:** 1 Laboratory of Molecular Neuroscience and Neurology, School of Life Sciences, Tokyo University of Pharmacy and Life Sciences, Hachioji, Tokyo 192-0392, Japan; 2 Tokyo Institute of Technology, School of Life Science and Technology, Yokohama, 226-8501, Japan

**Keywords:** Fragile X syndrome, Psychiatric disease, Startle response, Centaurin

## Abstract

The neural mechanisms of psychiatric diseases like autism spectrum disorder and schizophrenia have been intensively studied, and a number of candidate genes have been identified. However, the relationship between genes and neural system functioning remains unclear. Model organisms may serve as a powerful tool for addressing this question due to the availability of established genetic tools. Here, we report prepulse inhibition (PPI) in *Drosophila* larvae for the first time. PPI is a neurological phenomenon found in humans and other organisms and is used in the diagnosis of schizophrenia and other psychiatric disorders. A weaker prestimulus (prepulse) inhibits the reaction to a subsequent strong, startling stimulus (pulse). Using the larval startle response to the buzz of a predator (wasp), we examined PPI in wild-type flies and two mutants: an *fmr1* mutant, which is implicated in Fragile X syndrome, and a *centaurin gamma 1A* (CenG1A) mutant, which is associated with GTPase, PH, ArfGAP, and ANK domains and implicated in autism. Both mutants showed decreased PPI, whereas, interestingly, double mutants showed substantial PPI. The PPI phenomenon described here can provide a useful tool for the study of neural mechanisms of synaptic modification and psychiatric diseases.

## Abstract (precis)

**Summary:** Prepulse inhibition (PPI) deficiency is observed in various psychiatric disorders. Here we demonstrate PPI in *Drosophila* and its association with psychiatric disease-related genes.

## INTRODUCTION

When a startle stimulus is preceded by a weaker non-startle prestimulus, the robust startle response is inhibited. This innate phenomenon is known as prepulse inhibition (PPI) and is found in many organisms including humans, mice, and the invertebrate model organism *Tritonia diomedea* (Johansson et al., 1995; Mongeluzi et al., 1998; Braff et al., 2001; Geyer et al., 2001; Winslow et al., 2002;). PPI is deficient in schizophrenia and certain other neurological and psychiatric disorders (Braff et al., 2001; Geyer et al., 2001). Thus, PPI is of interest as a diagnostic symptom, and clarification of its neural mechanisms is important for studies of mental disorders.

It has been speculated that PPI is linked to the ability to filter out unnecessary information; in other words, it is linked to regulation of sensorimotor gating. Many efforts are underway to identify the neural mechanisms mediating PPI. Although these studies have identified relevant brain areas and neural networks (Braff et al., 2001; Fendt et al., 2001; Geyer et al., 2001), specific cellular mechanisms for vertebrate PPI have not yet been elucidated. By contrast, studies of the invertebrate model *T.* *diomedea* have described the cellular mechanism in detail and identified the axonal conduction block as a novel mechanism of PPI (Mongeluzi et al., 1998; Frost et al., 2003; Lee et al., 2012). However, genetic tools for this animal are not well established, preventing further molecular studies to clarify gene functions related to PPI. *Drosophila melanogaster* is a well-established invertebrate model for which many genetic tools are available. However, PPI has not been reported in *Drosophila*.

Here, we report PPI in the *Drosophila* larval startle response to a sound stimulus. Larvae display startle-freeze behaviour in response to the natural sound stimulus of wasps, a predator of larvae (Zhang et al., 2013). When a weaker non-startle stimulus preceded the startle stimulus, the freezing behaviour in response to the startle stimulus was decreased. To further demonstrate the efficacy of the experimental condition of PPI in *Drosophila*, we examined PPI in a Fragile X mental retardation 1 (*fmr1*) mutants. The gene *fmr1* is implicated in Fragile X syndrome (FXS) and is associated with clinically relevant behaviours including mental retardation, sleep disorders, hyperactivity, and autistic behaviour (Bakker and Oostra, 2003; Bear et al., 2004). Its homolog gene exists in *Drosophila* (Wan et al., 2000). Additionally, PPI was investigated in larvae with suppressed centaurin gamma 1A (CenG1A) function. CenG1A is a member of centaurin family and mammalian *centaurin gamma 2* is proposed to relate to psychiatric diseases (Wassink et al., 2005). These studies should shed light on the molecular and cellular mechanisms of PPI and psychiatric diseases.

## RESULTS

### PPI was found in the larval startle response

**Fig. 1.**
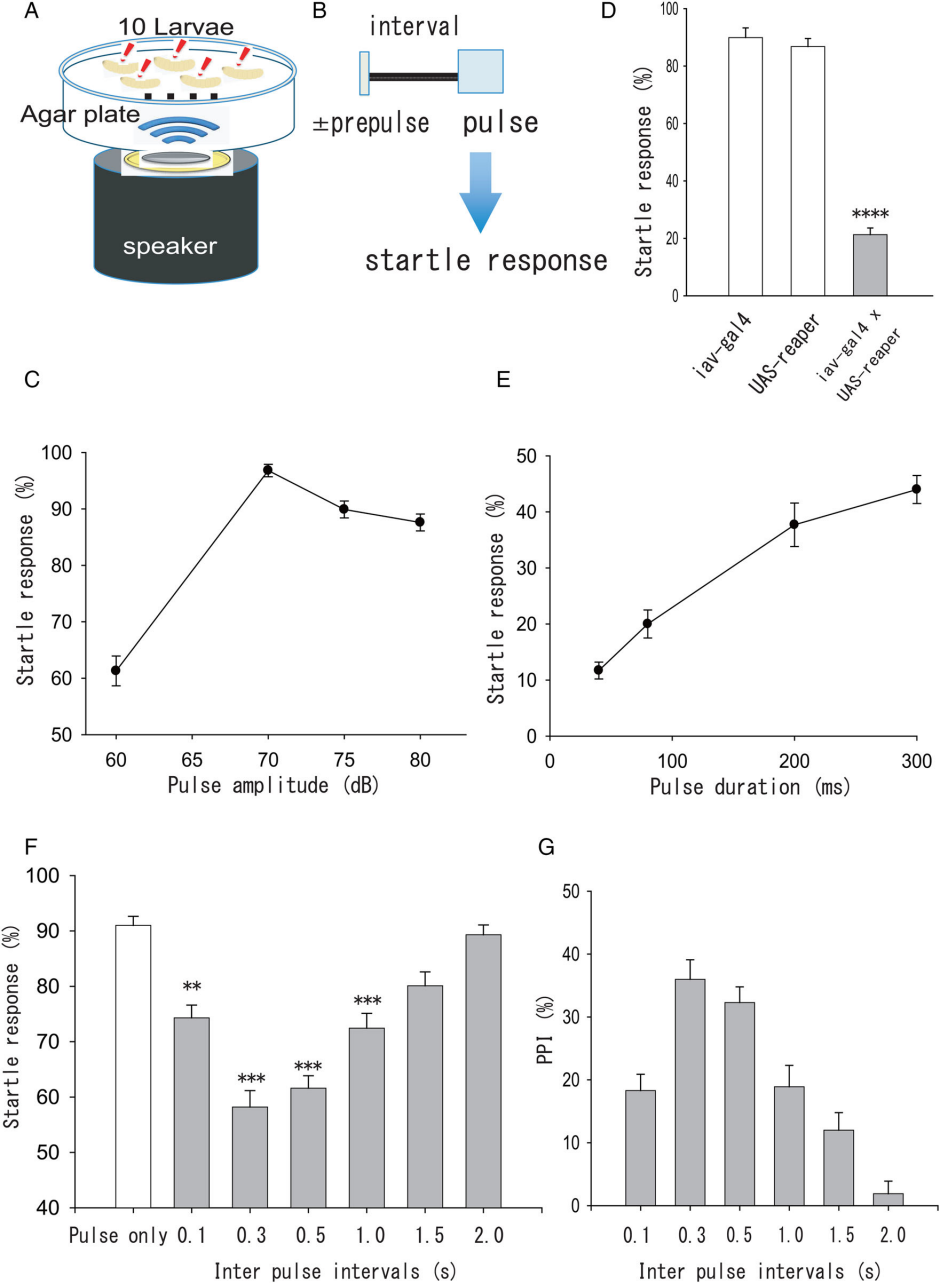
**The experimental protocol to test PPI using the startle response of *Drosophila* larvae to the sound of wasps.** (A) Schematic diagram of the system. An agar plate with 10 larvae was placed on the speaker. (B) Experimental protocol for PPI. Inter-pulse interval between the prepulse (40 ms duration) and pulse (500 ms duration) was changed from 0.1 s to 2.0 s. Larval behaviour was recorded, and the startle response to the pulse was analysed. Usually, this sequence was performed five times at 15 s intervals. (C) Startle response to various amplitudes of the pulse sound in wild-type flies (CS). A considerable percentage of the responses was observed at the smallest amplitude of the pulse. 60 dB: *n*=30 trials; 70 dB: *n*=25; 75 dB: *n*=15; 80 dB: *n*=15. (D) Suppression of iav neurons affected the startle response. *iav-gal4*: *n*=15 trials; *UAS-reaper*: *n*=15; *iav-gal4*×*UAS-reaper: n*=15, ****P<0.0001, ANOVA and post-hoc Scheffe's test. (E) The startle response was dependent on the duration of the sound stimulus. Duration at 40 ms was used as the prepulse. Data are from 15 trials of 30 larvae. (F) PPI was examined in CS. A 0.3 s inter-pulse interval was the most effective for inhibition of the startle response to the pulse. Pulse only: *n*=20 trials; 0.1 s interval: *n*=20; 0.3 s interval: *n*=20; 0.5 s interval: *n*=20; 1.0 s interval: *n*=20; 1.5 s interval: *n*=20; 2.0 s interval: *n*=20, **P<0.01, ***P<0.001, compared to the value of pulse only, ANOVA and post-hoc Scheffe's test. (G) PPI index calculated from data in F.

Although most animals show PPI, it has not been reported in *Drosophila*. To demonstrate and establish an experimental system of quantifying PPI in *Drosophila*, we used the larval startle response. Zhang et al. (2013) reported that larvae show a startle response to natural sounds of predators (wasps) or pure tones with a similar wavelength. First, we examined the larval startle response to wasp sounds in wild-type (CS) *Drosophila* larvae. Pulse sounds were made from wasp sound files as described in the Materials and Methods section. Although 1 s duration of the stimulus was used by Zhang et al. (2013), we used 500 ms duration, which was sufficient to induce the startle response. The agar plate with 10 CS larvae was placed on the speaker and delivered the sound stimulus (Fig. 1A). We calculated the percentage of larvae showing startle response in one trial and then averaged the percentages from several trials to calculate the startle response as described in the Materials and Methods section. We examined startle responses to pulse stimulations of 500 ms durations (Fig. S1A) with different amplitudes (Fig. 1C). More than 85% of larvae showed a startle response to sounds over 70 dB (see Movie 1 and Fig. 1C, 60 dB: 61.3%±2.9%, *n*=30 trials; 70 dB: 96.8%±1.1%, *n*=25 trials; 75 dB: 89.6%±1.3%, *n*=15; 80 dB: 87.6%±1.5%, *n*=15). We used the 75 dB setting for the pulse stimulation in the following experiments. It has been shown that startle responses are induced through chordotonal organs, which are sensory organs receiving sounds, vibrations, and other mechanical stimulations (Zhang et al., 2013; Ohyama et al., 2015; Jovanic et al., 2016). To confirm that the startle response we observed was also received through chordotonal (cho) organs, we suppressed the function of cho organs using cho neuron-specific ablation and examined the startle response. For cell-specific ablation, the reaper gene was specifically expressed and then apoptosis was induced in cho organ neurons using Gal4-upstream activation sequence (UAS) expression system (Brand and Perrimon, 1993). Inactive (iav)-Gal4 can be used for inducing cho neuron-specific expression (Zhang et al., 2013). As shown in Fig. 1D, cho organ neuron-specific ablation suppressed the startle response to the pulse compared with controls (Fig. 1D, *iav-gal4*:89.9%±3.4%, *n*=15 trials; *UAS-reaper*: 86.8%±2.3%, *n*=15; *iav-gal4*×*UAS-reaper*: 21.3%±2.3%, *n*=15, *P*<0.0001, ANOVA and post-hoc Scheffe's test), suggesting that the startle response we observed was induced through cho neurons. Next, we searched for a prepulse stimulation. Even sounds with the smallest amplitude used (around 60 dB) triggered a startle response in 61.3%±2.9% of larvae (Fig. 1C, *n*=30 trials). In PPI, a stimulus showing no response or a very low response is used as the prepulse. Therefore, it is difficult to make a sound function as a prepulse in PPI by changing the amplitude. We tested several sound sources with different waveforms and durations and identified candidate prepulse sounds for PPI (Fig. S1B,C). Increasing the duration of the prepulse candidates enhanced the startle response (Fig. 1E, 40 ms: 11.7%±1.5%, *n*=15 trials; 80 ms: 20.0%±2.5%, *n*=15; 200 ms: 37.7%±3.9%, *n*=15; 300 ms: 44.0%±2.5%, *n*=15). Increasing the duration of sound also increased its amplitude (Fig. S1B,C). We decided the sound at 40 ms duration as a prepulse because it induced the lowest response among all other tested durations (Fig. 1E). The waveforms of the prepulse had a gradually increasing amplitude, whereas those of the pulse started with a large amplitude (Fig. S1A,B, left panel). The power spectra of these sounds are the same, although the ratio of power around 300 Hz differs between the prepulse and pulse (Fig. S1A,B, right panel). Finally, we examined whether PPI was observed in larvae. Intervals between the prepulse and pulse were altered from 0.1 s to 2.0 s (see Fig. 1B). The startle response was inhibited by the precedent prepulse with 40 ms duration, and the effect of the prepulse was lost at intervals longer than 1.5 s (see Movie 2; Fig. 1F, pulse only: 91.0%±1.6%, *n*=20 trials; 0.1 s interval: 74.3%±2.3%, *n*=20, *P*=0.014; 0.3 s interval: 58.2%±3.0%, *n*=20, *P*<0.001; 0.5 s interval: 61.6%±2.3%, *n*=20, *P*<0.001; 1.0 s interval: 72.4%±2.7%, *n*=20, *P*<0.001; 1.5 s interval: 80.1%±2.5%, *n*=20, *P*=0.13; 2.0 s interval: 89.3%±1.8%, *n*=20, *P*=0.84, ANOVA and post-hoc Scheffe's test compared with the value of pulse only). We also tested a sound with 20 ms duration as a prepulse, but it did not show an effect on the startle response (data not shown). PPI index, which indicates the extent of inhibition of the response to the pulse by prepulse, was calculated from the difference between the response with and without prepulse as mentioned in the Materials and Methods section (Fig. 1G). The effect of prepulse was most effective at 0.3–0.5 s intervals (Fig. 1F,G). PPI was completely absent at 2.0 s intervals (Fig. 1F,G). The loss of the effect of prepulse with increasing intervals is a prominent characteristic of PPI. Thus, these results support that PPI is present in *Drosophila* and can be used in further experiments.

### PPI was lost in *fmr1*

**Fig. 2.**
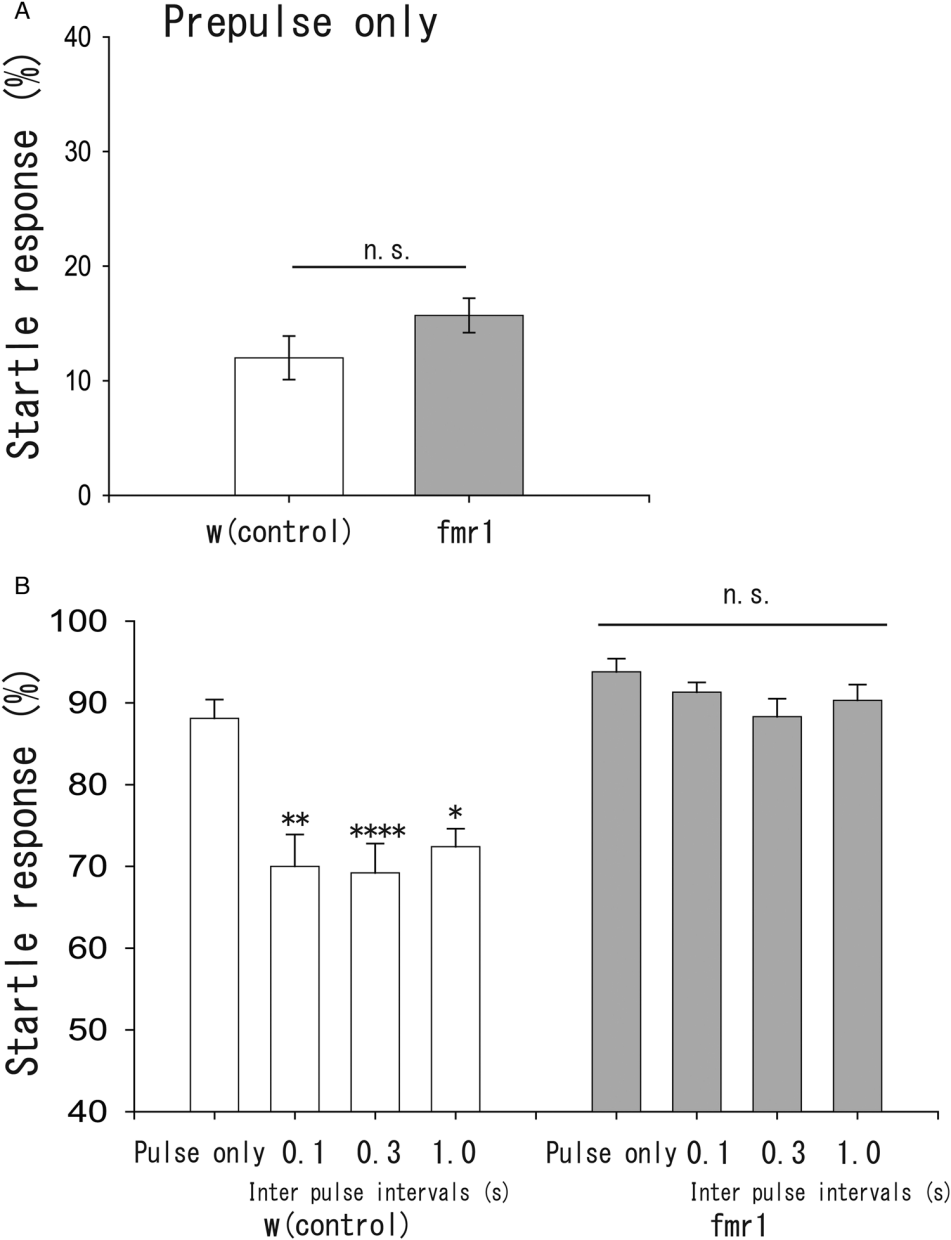
**PPI in *fmr1* mutant flies. For further validation of our PPI protocol, *w* (control) and *fmr1* flies were examined.** (A) The startle response to prepulse only. There was no difference in the startle response to the prepulse between controls and *fmr1* mutants (*w*, *n*=15 trials, *fmr1*, *n*=15 trials, n.s. not significant). (B) The startle response to pulse with and without prepulse. In *w*, the prepulse inhibited the startle response, but not in *fmr1* mutants. *w*, pulse only: *n*=25 trials; 0.1 s: *n*=15; 0.3 s: *n*=25; 1.0 s: *n*=20, *P<0.05, **P<0.01, ****P<0.0001, *fmr1* mutants, pulse only: *n*=20 trials; 0.1 s: *n*=15; 0.3 s: *n*=20; 1.0 s: *n*=15, *P*=0.2, ANOVA and post-hoc Scheffe's test, n.s. not significant.

It is well known that PPI is affected in patients with schizophrenia and autism and in animal models of these diseases (Braff et al., 2001; Geyer et al., 2001; Frankland et al., 2004). To further test the efficacy of our established PPI in larval behaviour, the FXS model of *Drosophila,* which has been extensively studied, was examined. *fmr1* is a human gene that codes for a protein called the Fragile X mental retardation protein (FMRP) (O'Donnell and Warren, 2002). Mutations in this gene can lead to FXS, which shows intellectual disability, developmental delays, other cognitive deficits, and autistic symptoms (Bhakar et al., 2012). FMRP is thought to be a transcription factor that is important for neural and cognitive development. In *Drosophila*, there is a homolog of *fmr1* with a similar function (Zarnescu et al., 2005; McBride et al., 2013). Therefore, we investigated PPI in *fmr1* mutants*.* The startle response to either a prepulse or pulse was not significantly different between control (*white*, *w*) and *fmr1* mutant flies, while the mean value of the response was slightly greater in *fmr1* mutants than in controls (*w*) (Fig. 2A, *w*: 12.0%±1.9%, *n*=15 trials; *fmr1*: 15.7%±1.5%, *n*=15, *P*=0.16, Student's *t*-test, Fig. 2B, pulse only, *w*: 88.1%±2.3%, *n*=25; *fmr1*: 93.8%±1.6%, *n*=20, *P*=0.08, Mann–Whitney *U*-test). We noticed that *fmr1* mutants showed an exaggerated startle response to pulse sounds (see Movie 3). On the other hand, PPI was suppressed in *fmr1* mutants. In the control, the startle response to the pulse was inhibited by the precedent prepulse, indicating the presence of PPI (Fig. 2B, 0.1 s interval: 70.0%±3.9%, *n*=15 trials, *P*=0.0098; 0.3 s interval: 69.2%±3.6%, *n*=25 trials, *P*<0.0001; 1.0 s interval: 72.4%±2.2%, *n*=20 trials, *P*=0.02, compared with the value of pulse only, ANOVA and post-hoc Scheffe's test). In contrast, the startle response was not significantly decreased by the prepulse in *fmr1* mutants (Fig. 2B, 0.1 s interval: 91.3%±1.2%, *n*=15 trials; 0.3 s interval: 88.3%±2.2%, *n*=20 trials; 1.0 s interval: 90.3%±1.9%, *n*=15 trials, *P*=0.20, ANOVA, also see Movie 4). These results suggest that PPI was affected in *fmr1* mutants. Thus, PPI could be used to test other mutations possibly related to psychiatric disorders.

### PPI in CenG1A mutants and interaction between CenG1A and FMRP

**Fig. 3.**
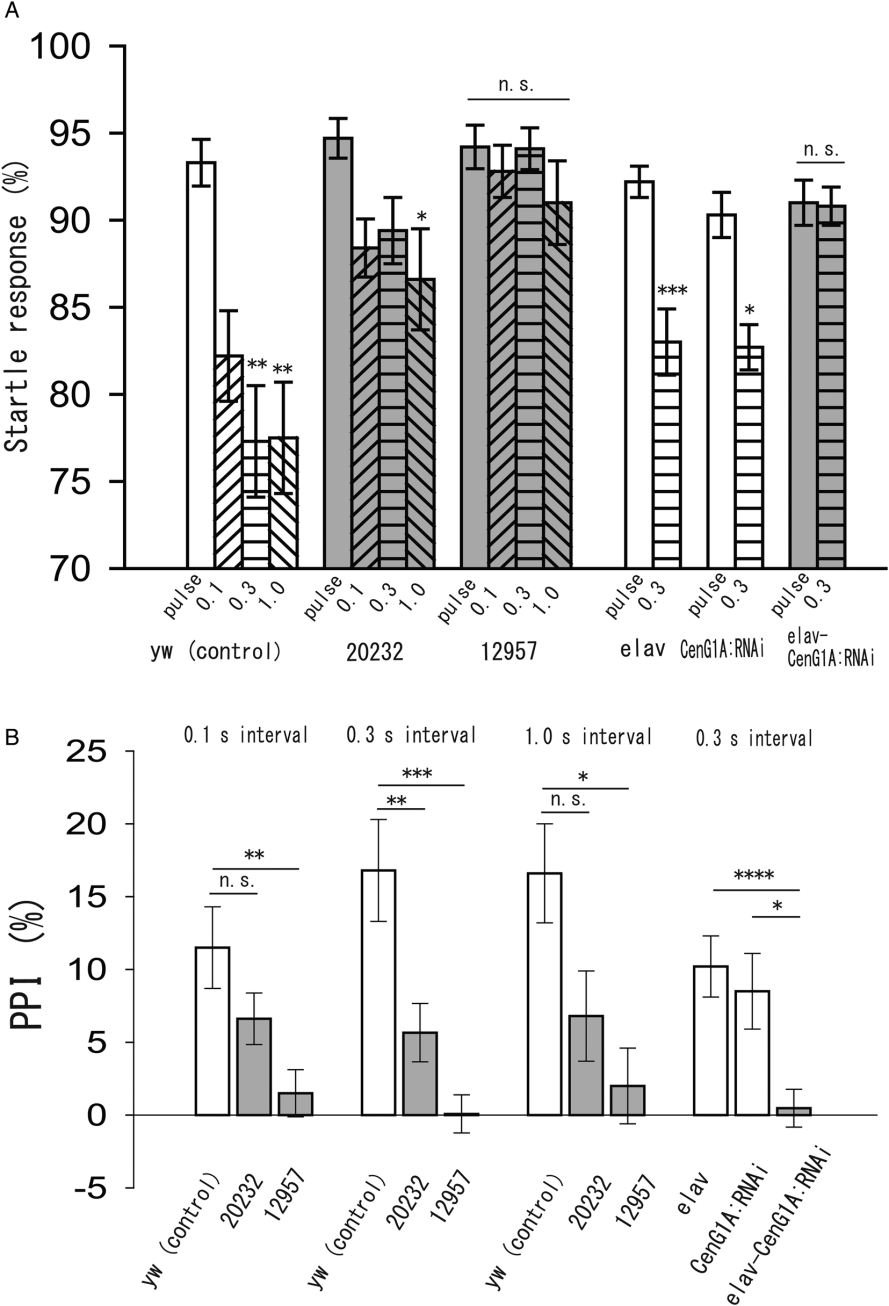
**PPI in larvae with suppressed CenG1A function.** (A) Left group; the startle response to pulse-only and prepulse-pulse stimulations at 0.1 s, 0.3 s, and 1.0 s intervals in control (*yw*, white columns) and transposable P-element insertion lines (*20232* and *12957*, grey columns). Right group; the startle response to pulse-only and prepulse-pulse stimulation at 0.3 s interval in elav (white columns), CenG1A:RNAi (white columns), and elav-CenG1A:RNAi (grey columns), which were larvae with suppressed CenG1A function in neurons using the Gal4-UAS expression system. Prepulse effectively inhibited the response in controls, but not in 12957 and elav-CenG1A:RNAi. In *20232*, the prepulse was partially effective (left group, *yw*: pulse only, *n*=25 trials; 0.1 s interval, *n*=25; 0.3 s interval, *n*=25; 1.0 s interval, *n*=25; *20232*: pulse only, *n*=25; 0.1 s interval, *n*=25; 0.3 s interval, *n*=25; 1.0 s interval, *n*=15; *12957*: pulse only, *n*=25; 0.1 s interval, *n*=25; 0.3 s interval, *n*=25; 1.0 s interval, *n*=10, *P<0.05, **P<0.01, ANOVA and post-hoc Scheffe's test. Right group, elav: pulse only, *n*=35; 0.3 s interval, *n*=35; CenG1A:RNAi: pulse only, *n*=25; 0.3 s interval, *n*=30; elav-CenG1A:RNAi: pulse only, *n*=35; 0.3 s interval, *n*=35, *P<0.05, ***P<0.001, Mann–Whitney *U*-test, n. s. not significant). (B) PPI was affected by decreased CenG1A function. At 0.3 s, PPI was significantly decreased in both *20232* and *12957*. At 0.1 s and 1.0 s inter-pulse intervals, only *12957* showed reduction of PPI. PPI was significantly suppressed in elav-CenG1A:RNAi compared with elav. PPI was calculated from data in Fig. 3A. *P<0.05, **P<0.01, ***P<0.001, ****P<0.0001, n.s. not significant, ANOVA and post-hoc Scheffe's test.

We have previously reported that CenG1A is a negative regulator of neural transmission and may have an important role in neural function (Homma et al., 2014). It has been suggested that FMRP negatively regulates the transcription of centaurin gamma 1 (CenG1) in mice, and thus CenG1 is related to FXS (Darnell et al., 2011; Gross et al., 2010; Sharma et al., 2010). Here, we tested PPI in transposable P-element-inserted CenG1A mutants (*20232* and *12957*) used in a previous study (Homma et al., 2014) and larvae with suppressed CenG1A expression in all neurons using RNA interference (RNAi) method. Specific expression of RNAi construct for CenG1A was induced by the GAL4-UAS expression system (Brand and Perrimon, 1993). The elav and CenG1A:RNAi lines were used as Gal4 and UAS lines, respectively. elav-CenG1A:RNAi was the line with suppressed CenG1A function in all neurons. There were no significant differences in the startle response to the pulse between *yellow white* (*yw*) and mutants (Fig. 3A, *yw*: 92.9%±1.4%, *n*=25 trials; *20232*: 94.7%±1.1%, *n*=25; *12957*: 94.2%±1.3%, *n*=25, *P*=0.73, Kruskal–Wallis ANOVA) and also among elav, CenG1A:RNAi, and elav-CenG1A:RNAi lines (Fig. 3A, elav: 92.2%±0.9%, *n*=35; CenG1A:RNAi: 90.3%±1.3%, *n*=25; elav-CenG1A:RNAi: 91.0%±1.3%, *n*=35, *P*=0.81, Kruskal–Wallis ANOVA). We noted that there was a slight difference in the startle response between these two groups, probably because of the difference in genetic backgrounds. On the other hand, PPI was affected by inhibiting CenG1A function. In the control lines, *yw*, elav, and CenG1A:RNAi, precedent prepulse significantly suppressed the startle response, suggesting the presence of PPI (Fig. 3A, *yw*: 0.1 s interval, 82.2%±2.6%, *n*=25 trials, *P*=0.058; 0.3 s interval, 77.3%±3.2%, *n*=25 trials, *P*=0.0016; 1.0 s interval, 77.5%±3.2%, *n*=25 trials, *P*=0.0018, compared with the values of pulse only, ANOVA and post-hoc Scheffe's test, elav: 0.3 s interval, 83.0%±1.9%, *n*=35 trials, *P*<0.001; CenG1A:RNAi: 0.3 s interval, 82.7%±1.3%, *n*=30 trials, *P*=0.016, Mann–Whitney *U*-test). On the contrary, precedent prepulse could not inhibit the response in the *P*-element-inserted mutants, *12957*, and larvae with suppressed CenG1A expression in all neurons (elav-CenG1A:RNAi) (Fig. 3A, *12957*: 0.1 s interval, 92.8%±1.5%, *n*=25 trials; 0.3 s interval, 94.0%±1.2%, *n*=25 trials; 1.0 s interval, 91.0%±2.4%, *n*=10 trials, *P*=0.57, ANOVA, elav-CenG1A:RNAi: 0.3 s interval, 90.8%±1.1%, *n*=35 trials, *P*=0.43, Mann–Whitney *U*-test). In the other P-element inserted mutants, *20232*, the prepulse was slightly effective on inhibiting the startle response (Fig. 3A, *20232*: 0.1 s interval, 88.4%±1.7%, *n*=25 trials, *P*=0.09; 0.3 s interval, 89.4%±1.9%, *n*=25 trials, *P*=0.19; 1.0 s interval, 86.6%±2.9%, *n*=15 trials, *P*=0.04, compared with the values of pulse only, ANOVA and post-hoc Scheffe's test). PPI index was calculated from data in Fig. 3A and compared within genotypes. PPI was suppressed in CenG1A mutants (Fig. 3B, 0.1 s interval, *yw*: 11.5%±2.8%, *20232*: 6.6%±1.8%, *12957*: 1.5%±1.6%, *P*=0.27 for *yw* versus *20232, P*=0.0059 for *yw* versus *12957*; 0.3 s interval, *yw*: 16.8%±3.5%, *20232*: 5.7%±2.0%, *12957*: 0.08%±1.3%, *P*=0.0073 for *yw* versus *20232, P*<0.001 for *yw* versus *12957*; 1.0 s interval, *yw*: 16.6%±3.43%, *20232*: 6.8%±3.1%, *12957*: 2.0%±2.6%, *P*=0.12 for *yw* versus *20232, P*=0.033 for *yw* versus *12957*, ANOVA and post-hoc Scheffe's test). The inhibition of PPI was clearer in the *12957* mutant compared with that in the *20232* mutant. Moreover, PPI index in larvae with suppressed CenG1A expression (elav-CenG1A:RNAi) was significantly lower compared with that in their control lines (Fig. 3B right, elav: 10.2%±2.1%, CenG1A:RNAi, 8.5%±2.6%, elav-CenG1A:RNAi, 0.47%±1.3%, *P*<0.0001, elav versus elav-CenG1A:RNAi, *P*=0.024, CenG1A:RNAi versus elav-CenG1A:RNAi, ANOVA and post-hoc Scheffe's test). Taken together, these results suggest that CenG1A plays a pivotal function in the modulation of the neural system related to PPI.

**Fig. 4.**
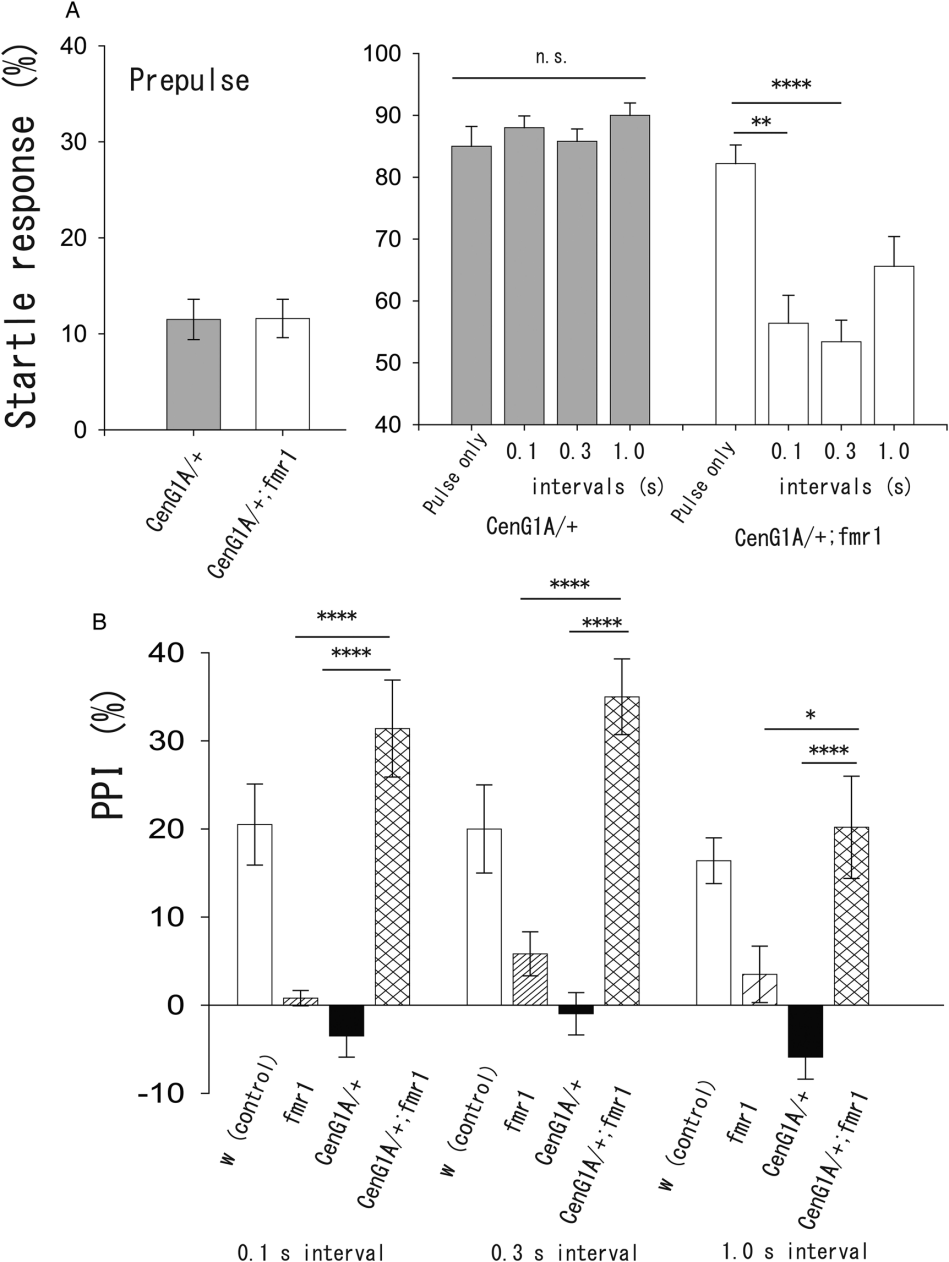
**Interaction between CenG1A and FMRP.** (A) The startle responses to prepulse (left) and pulse with or without prepulse (right) in heterozygotes of CenG1A (*CenG1A/+*), and heterozygotes of CenG1A under *fmr1* background (*CenG1A/+; fmr*). Left, *CenG1A/+*: *n*=10 trials; *CenG1A/+; fmr1*: *n*=10 trials, right, *CenG1A/+*: pulse only, *n*=20 trials; 0.1 s interval, *n*=10; 0.3 s interval, *n*=20; 1.0 s interval, *n*=10; *CenG1A/+; fmr1*: pulse only, *n*=25 trials; 0.1 s interval, *n*=10; 0.3 s interval, *n*=30; 1.0 s interval, *n*=10, n.s. not significant, **P<0.01, ****P<0.0001, ANOVA and post-hoc Scheffe's test. (B) PPI at 0.1 s intervals (left), 0.3 s interval (middle), and 1.0 s intervals (right) in *white* (*w*), *fmr1*, heterozygotes of CenG1A (*CenG1A/+*), and heterozygotes of CenG1A under *fmr* background (*CenG1A/+; fmr1*). As shown in Fig. 3, suppression of CenG1A function in heterozygotes led to the loss of PPI. However, double CenG1A and *fmr1* mutants showed substantial PPI. PPI in *CenG1A/+; fmr1* was significantly higher than in *fmr1* and *CenG1A/+* at all intervals. PPI index was calculated from data in Figs 2B and 4A. *P<0.05, ****P<0.0001, ANOVA and post-hoc Scheffe's test.

Recently, Gross et al. (2015) reported that increased expression of phosphoinositide-3 kinase enhancer PIKE mediates deficits in synaptic plasticity and behaviour in FXS model of mice and flies. PIKE is another name for CenG1 in mammals and CenG1A in *Drosophila*. Gross et al. (2015) showed that genetic reduction of CenG1A rescued morphological defects in the mushroom body, a central region of the fly brain, and impaired short-term memory in FXS flies. Therefore, we examined PPI in FXS larvae with genetically decreased expression of CenG1A. There were no notable differences in the startle response to the prepulse or pulse only between heterozygotes of CenG1A (*CenG1A*/+) and FXS larvae with genetically reduced expression of CenG1A (*CenG1A*/+; *fmr1*) (Fig. 4A, prepulse only, *CenG1A*/+: 11.5%±2.1%, *n*=10, *CenG1A*/+; *fmr1*, 11.6%±2.0%, *n*=10; pulse only, *CenG1A*/+: 85.0%±3.2%, *n*=20, *CenG1A*/+; *fmr1*: 82%±3.0%, *n*=25). These values were similar with that of *w* in Fig. 2A and B (Fig. 2A, *w*: 12.0%±1.9%; Fig. 2B, *w*: 88.1%±2.3%). As expected according to the results shown in Fig. 3, the genetic reduction of CenG1A suppressed the inhibitory effect of prepulse on the startle response to the pulse (Fig. 4A, *CenG1A*/+: 0.1 s interval, 88.0%±1.9%, *n*=10; 0.3 s interval, 85.8%±2.0%, *n*=20; 1.0 s interval, 90.0%±2.0%, *n*=10, *P*=0.63, ANOVA). However, FXS larvae with genetic reduction of CenG1A expression showed the inhibition of the startle response to the pulse induced by the precedent prepulse, suggesting the presence of PPI (Fig. 4A, *CenG1A*/+; *fmr1*: 0.1 s interval, 56.4%±4.5%, *n*=10, *P*=0.0018; 0.3 s interval, 53.4%±3.5%, *n*=30, *P*<0.0001; 1.0 s interval, 65.6%±4.8%, *n*=10, *P*=0.086, compared with the value of pulse only, ANOVA and post-hoc Scheffe's test). We further compared PPI index among the *w*, *fmr1*, *CenG1A*/+, and FXS larvae with genetically decreased expression of CenG1A (*CenG1A*/+; *fmr1*) (Fig. 4B). We found that the defect in PPI in CenG1A heterozygotes and *fmr1* mutants was rescued by the combination of these two genotypes. PPI was successively observed in homozygous *fmr1* mutant flies that were heterozygous for a mutant allele of CenG1A (Fig. 4B, 0.1 s interval, *w*: 20.5%±4.6%, *n*=15 trials; *fmr1*: 0.8%±0.87%, *n*=15; *CenG1A*/+: −3.5%±2.4%, *n*=10; *CenG1A*/+; *fmr1*, 31.4%±5.5%, *n*=10, *P*<0.0001 for *fmr1* versus *CenG1A*/+; *fmr1, P*<0.0001 for *CenG1A*/+ versus *CenG1A*/+; *fmr1*; 0.3 s interval, *w*: 20.0%±5.0%, *n*=20 trials; *fmr1*: 5.8%±2.5%, *n*=20, *CenG1A*/+: −0.97%±2.4%, *n*=20; *CenG1A*/+; *fmr1*, 35.0%±4.3%, *n*=30, *P*<0.0001 for *fmr1* versus *CenG1A*/+; *fmr1, P*<0.0001 for *CenG1A*/+ versus *CenG1A*/+; *fmr1*; 1.0 s interval, *w*: 16.4%±2.6%, *n*=20 trials; *fmr1*: 3.5%±3.2%, *n*=10; *CenG1A*/+: −5.9%±2.5%, *n*=10; *CenG1A*/+; *fmr1*, 20.2%±5.8%, *n*=10, *P*=0.038 for *fmr1* versus *CenG1A*/+; *fmr1, P*<0.0001 for *CenG1A*/+ versus *CenG1A*/+; *fmr1,* ANOVA and post-hoc Scheffe's test). These results suggest the existence of interaction between CenG1A and FMRP, possibly the regulation of transcription of CenG1A through FMRP, as suggested in previous studies (Darnell et al., 2011; Gross et al., 2010, 2015; Sharma et al., 2010). In addition, the expression level of CenG1A may have an important role in synaptic function and modulation in the neural mechanisms of PPI.

## DISCUSSION

PPI is a neurological phenomenon that has attracted much interest due to its deficiency in schizophrenia and other neurological and psychiatric disorders (Braff et al., 2001; Geyer et al., 2001). PPI in *Drosophila* may serve as a useful model for further studies of psychiatric and neurological disorders. PPI, in our study, satisfied the following two features, which are generally observed in PPI of other animals. First, PPI was dependent on time intervals between the prepulse and pulse. Second, PPI was decreased in *fmr1* mutants; decreased PPI has previously been shown in FXS patients and PPI also affected in *fmr1* knockout mice (Frankland et al., 2004; Hessl et al., 2009). Thus, PPI presented here shares features with the PPI paradigm in mammals and other organisms.

Sound signals mediate important information in contexts ranging from courtship to detection of predators or prey. *Drosophila* larvae were found to respond to the sound of wasps with several types of robust startle behaviour (Zhang et al., 2013). Although we used the sound as stimulation for the induction of the startle response, it is likely that this behaviour was induced by substrate-borne vibrations and not by vibrations of the air, because the behaviour became weak if the agar plate was kept away from the speaker. The startle behaviour observed in the present study has been previously reported in Zhang et al. (2013) and Jovanic, et al. (2016). Although the former report named this behaviour to be sound-induced, it is clear that mechanosensory stimulations through cho organs were responsible for the behaviour in both reports. Here, we confirmed that the behaviour we observed was triggered by mechanosensory stimulations through chordotonal organs (Fig. 1D). In fact, whether this behaviour belongs to one induced through hearing or not was not a focus point in the present study because it is well known that PPI is observed in any sensory modalities, such as hearing, touch, and vision (Ison and Hammond, 1971; Graham, 1975; Pinckney, 1976).

From the definition of a prepulse used in PPI, the prepulse must be a weak stimulus that mostly does not induce the startle response. The pulse sounds that induced robust startle responses in our study were loud at the beginning (Fig. S1A). Due to the limitations of our facility, the regulation of the amplitude did not work well in finding a sound that functioned as a prepulse. Instead, another sound source, as shown in Fig. S1B, was used and the prepulse was selected as a sound with a 40 ms duration, which induced less than 15% of the maximum startle response (Fig. 1E). Sounds shorter than 40 ms were not as effective as prepulses. Startle responses were effectively attenuated in wild-type larvae when prepulses preceded pulse sounds by 0.3 s (Fig. 1F). Effective intervals ranged from 0.1 s to 1.0 s, which are similar to ranges reported for PPI in *T.* *diomedea* (0.12 s–2.5 s, Mongeluzi et al., 1998; Frost et al., 2003). This time course is longer than most – but not all – examples of PPI in vertebrates including humans, which typically range from 30 ms to 300 ms (normally 60–120 ms). In other cases, such as tactile-elicited PPI of the human knee-jerk reflex or the rat startle reflex, the effect of prepulse lasts up to the 2 s intervals (Bowditch and Warren, 1890; Pinckney, 1976). These differences may reflect the differences in neural pathways related to the startle response in each animal. As prepulse is known to be effective in any modality, it would be interesting to examine whether a modality such as temperature sensation would work as a prepulse.

We noted that there were variations in the values of startle response and PPI index among several control lines (CS, *w*, *yw*, elav, and CenG1A:RNAi). These differences could be due to the differences in genetic backgrounds. In any case, the presence of PPI is very solid in these control lines. Further, the deviation of the score was rather high. To overcome these issues, the automatic quantification of the behaviour using software such as FIMTrack (Risse et al., 2017) will be very useful in future studies.

The neural pathway related to the larval startle response to sound stimuli has been recently identified (Zhang et al., 2013; Ohyama et al., 2015; Jovanic et al., 2016; Kristan, 2017). Cho organs are the main hearing organs for insects, and larval cho neurons are responsible for receiving sound stimuli and other mechanical stimulation (Zhang et al., 2013; Ohyama et al., 2015; Jovanic et al., 2016). Recent functional connectomics research has revealed the neural pathways that induce the startle behaviours through mechanical stimulation. These behaviours include cessation of crawling and either bending or shortening (called hunching), which were also observed in this study. The downstream circuit of cho neurons consists of secondary projection neurons and local interneurons, which function as both feedforward and feedback inhibitions. It is likely that inhibition of startle behaviours with the prepulse relate to synaptic modulation in this neural circuit. It would be both interesting and important to identify the synaptic site inhibited by the prepulse and the cellular mechanisms of PPI.

CenG1A is a potential target for phosphatidyl-inositol 3,4,5-triphosphate kinase (PI3K), which interacts with the ADP ribosylation factor (Arf) as an Arf-GTPase-activating protein (Jackson et al., 2000). Recently, we reported that CenG1A has a role in synaptic function as a negative regulator of neurotransmitter release (Homma et al., 2014). Interestingly, the mammalian homolog, *centaurin gamma 2*, is reported to be an autism susceptibility gene (Wassink et al., 2005). *CenG1* mRNA is associated with FMRP, leading to increased CenG1 protein levels in *fmr*1 knockout mice (Darnell et al., 2011; Gross et al., 2010; Sharma et al., 2010). A recent study proposed a hypothesis that increased expression of PIKE is a key mediator of deficits in synaptic plasticity and behaviour in mouse and fly models of FXS (Gross et al., 2015) although it is not clarified if *CenG1A* mRNA is truly a target of FMRP in flies. Our results in which the heterogenetic mutation of CenG1A rescued PPI suppression in *fmr1* mutants support this hypothesis. Additionally, the suppression of CenG1A expression resulted in a loss of PPI, suggesting that the appropriate expression level of CenG1A is a key mediator in the modulation of sensory processing. The relationship between CenG1A and FXS could be more clearly shown by quantifying the expression levels of CenG1A in several fly genotypes. Further studies will shed new light on the function of CenG1A in psychiatric defects.

In conclusion, we established a PPI test in *Drosophila* and demonstrated its potential. We believe that this PPI test will be useful for further studies of the function of genes related to psychiatric diseases, such as CenG1A.

## MATERIALS AND METHODS

### Flies

*D. melanogaster* were reared at room temperature (22°C). Only larvae used in the experiment described in Fig. 1D were reared at 28°C to increase the expression of Gal4. *Canton-S* (CS) was used as the wild-type strain; *yellow-white* (*yw*) or *white* (*w*) were used as the control strain for comparison with mutants. Transposable P-element insertion lines *20232* or *12957* (*cenG1A* mutants, used in Homma et al., 2014), *CenG1A*^EY01217^/*cyo* (CenG1A allele), and *fmr1*^Δ50M^ (*fmr1* mutant) were obtained from Bloomington Stock Center (Bloomington, USA). *20232* and *12957* were hypomorphic mutants and homozygotes were viable. We made *CenG1A*/*+*; *fmr1* from *CenG1A*^EY01217^/*cyo* and *fmr1*^Δ50M^, as described in a previous study (Gross et al., 2015). Almost all homozygous *CenG1A*^EY01217^ mutants in our stocks died before the third instar larval stage. A null CenG1A mutant was made by homologous recombination. Most homozygous *CenG1A* null mutants also died before the third instar larval stage. Cell-specific expression was achieved using the GAL4-UAS expression system (Brand and Perrimon, 1993). *elav-Gal4* (Luo et al., 1994, provided by Dr A. Nose) was used to induce expression in neurons. *iav-Gal4* (kindly provided by Dr C. Montell) induces the expression in cho organ neurons. UAS-reaper was obtained from Bloomington Stock Center. The expression of reaper induces apoptosis and ablates the specific neural function (Kiss et al., 2013). The RNA interference (RNAi) line *UAS-31811R-2* was obtained from the National Institute of Genetics (NIG). To induce cell-specific CenG1A knockdown, *UAS-31811R-2* was crossed with *elav-Gal4* (elav-CenG1A:RNAi). CS flies were crossed with either the GAL4 or UAS line as control animals (elav or CenG1A:RNAi). About 60 flies were placed in a vial for half a day to lay eggs. Larvae were collected 4 d later – which were living in the food and had not yet reached the wandering stage – and were used for the experiments.

### Behavioural analysis

Larvae were collected from fly food, washed with distilled water, and placed on plates (diameter 3.5 cm) covered with 5.3 ml of 2% agarose (Ina Food Company, Nagano, Japan). 10 larvae were used for one plate. Next, the agarose plate with 10 larvae was placed on a speaker (EZEEY T10, Amazon, Japan, see Fig. 1A). Larval behaviour during the experiments was recorded using a digital video camera (GZ-E345-V, Victor, Yokohama, Japan). Sounds that induced a strong larval startle response (pulse, 500 ms duration) and weak response (prepulse, 40 ms duration) were made from natural recordings of wasps on the Jungle Walk website, described in Zhang et al., 2013 and modified using WavePad software (NCH Software, Greenwood Village, USA). The pulse sound was obtained from a part of the wasp3 recording, whereas the prepulse sound was obtained from the beginning of the wasp3 recording. The wave forms of the sounds and power spectra of these waves are shown in Fig. S1.

We presented the sound stimulus five times at 15 s intervals to 10 larvae on the plate. These numbers of repetitions and intervals were determined by observing larval response in order to maintain a similar response rate. Because of this limitation, pulse-only stimulations and prepulse/pulse stimulations were tested separately. However, we confirmed that PPI was observed when the same larvae were delivered pulse-only and prepulse/pulse stimulations. Larval response to the pulse was scored as two points (strong startle response), one point (slight startle response), or zero points (no startle response). A score of two was given if the larvae exhibited startle behaviour including mouth-hook retraction, excessive turning, and/or backward locomotion, in response to the pulse sound stimulation. If it was difficult to discriminate whether the observed startle behaviour was a sequential behaviour starting before the sound stimulus or pulse-evoked response, we scored it as one point. Simple brief pausing for less than 1 s was scored as zero points. We then calculated the total points for one sound (one trial) in 10 larvae and the ratio against the full score (2 points×10 larvae=20 points). This ratio was defined as the startle response value for one trial. We removed larvae that did not move at all. If more than 3 larvae did not move, we discarded the data for that trial. To determine the startle response value for each condition, the startle response value was averaged across 15–25 trials using 30–50 larvae on 3–5 plates. To quantify prepulse inhibition (PPI) in one trial, the difference between the averaged value of the startle response to the stimulus without the prepulse (=X) and the value of the startle response in one trial (=Y) was divided by the averaged value of the startle response to the stimulus without the prepulse (PPI=(Y−X)/X). The PPI value for one trial was averaged over 15–25 trials using 30–50 larvae on 3–5 plates. We used the same larvae for only five trials of the same stimulus to avoid habituation. In some cases, the results were confirmed by the independent scoring of larval behaviour by more than two observers. Statistical analysis was performed using analysis of variance (ANOVA) and post-hoc Scheffe’s test for more than three samples. For comparisons involving two samples, we used the Student's *t*-test or Mann–Whitney *U*-test. Data were shown as the mean±s.e.m. Sound amplitude was roughly monitored using a simple application (Decibel X, SkyPaw) on a smartphone. The sound amplitude before stimulations was approximately 50 dB.

## Supplementary Material

Supplementary information
