# Supplementary material for: Prepulse inhibition in Drosophila melanogaster larvae
Source: Biol Open. 2018 Sep 15;7(9):bio034710. doi: 10.1242/bio.034710 (PMC6176951; doi:10.1242/bio.034710)
Supplement: Supplementary information [file biolopen-7-034710-s1.pdf]

Supplemental materials and methods

The wave forms of sound stimuli were shown by the WavePad software (NCH Software, Greenwood Village, CO, USA). Fourier transform analysis and figures of power spectrum was performed by the Matlab software (The MathWorks, Inc., MA, USA). Sample videos were made from videos used for data. For the better understanding, we used a part of the window to magnify the image using iMovie and Windows Live Movie Maker. Noises from the environment during intervals were cut off by Adobe Premiere Pro software (Adobe Systems Inc., CA, USA).

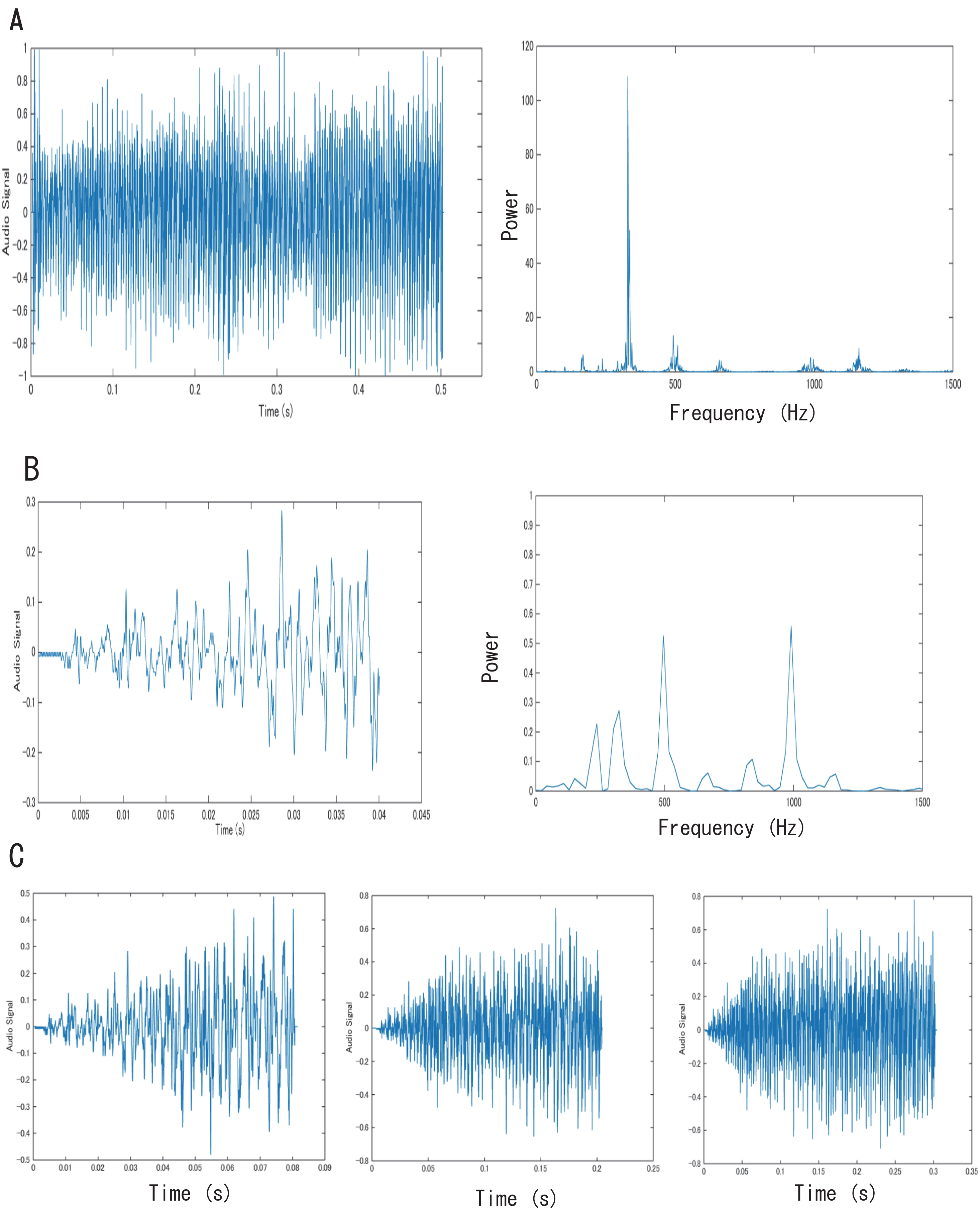

Biology Open • Supplementary information

Fig. S1. Wave forms and power spectra of the sounds used. (A) The sound used for a pulse. Left, wave form analyzed by the WavePad software. Right, power spectrum analyzed by Matlab. A wave content of approximately 300 Hz was the most prominent. (B) The sound used for the prepulse. Left, wave form analyzed by the WavePad software. Right, power spectrum analyzed using Matlab. Approximately 300 Hz, 500 Hz, and 1000 Hz wave contents were the most prominent. (C) Wave forms used in Fig. 1E. Left, 80 ms duration, middle, 200 ms duration, 300 ms duration.

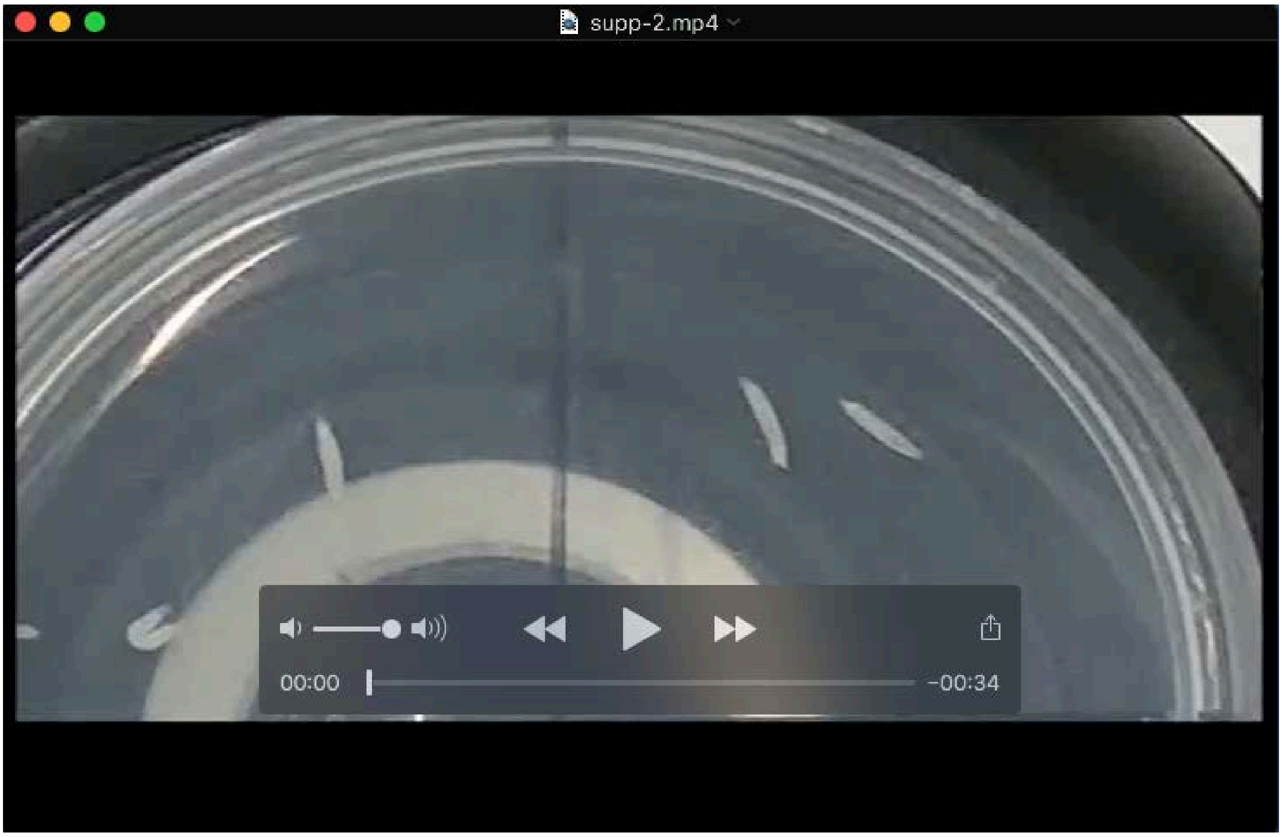

Movie S1. The startle response to the pulse. We provided the score of some larvae for a better understanding.

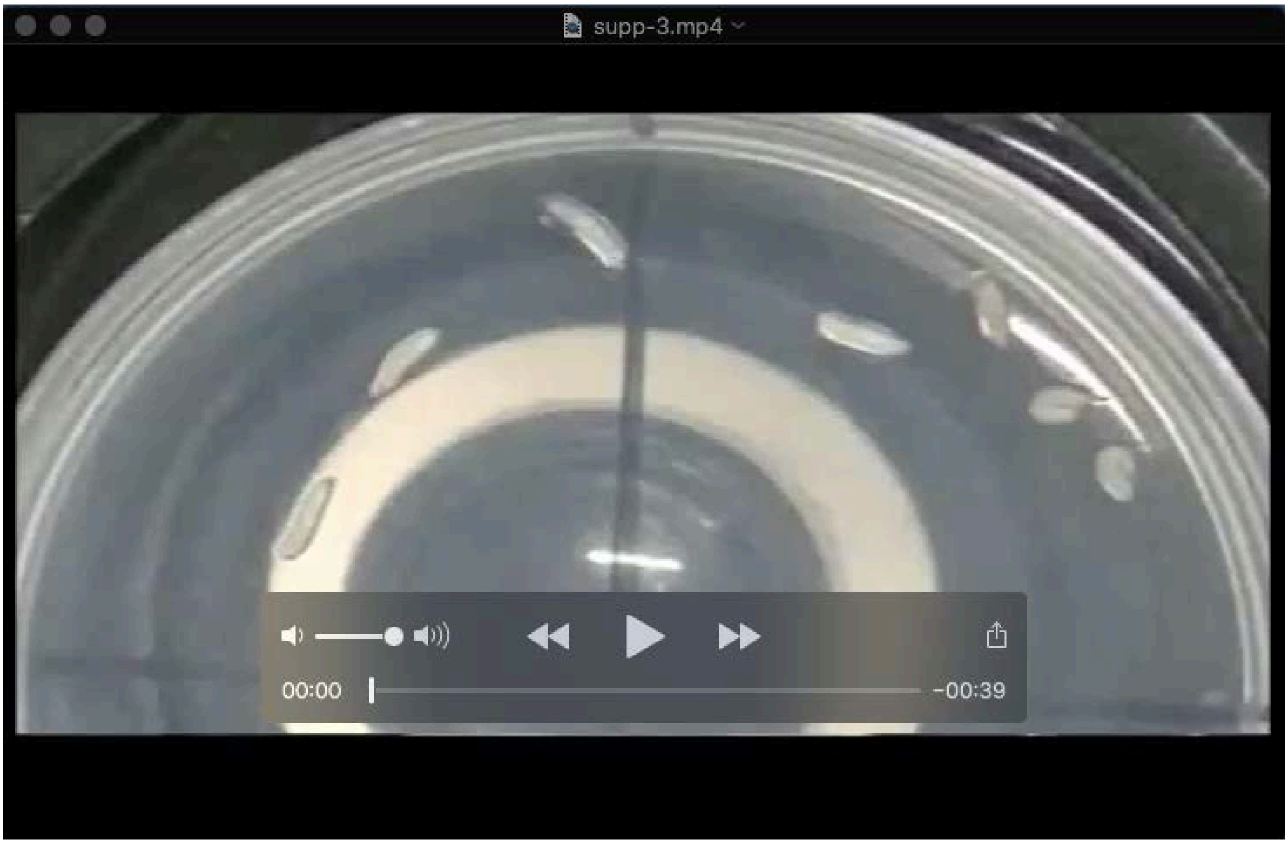

Movie S2. The startle response to the pulse with the precedent prepulse at intervals of 0.3 s. We provided the score of some larvae for a better understanding.

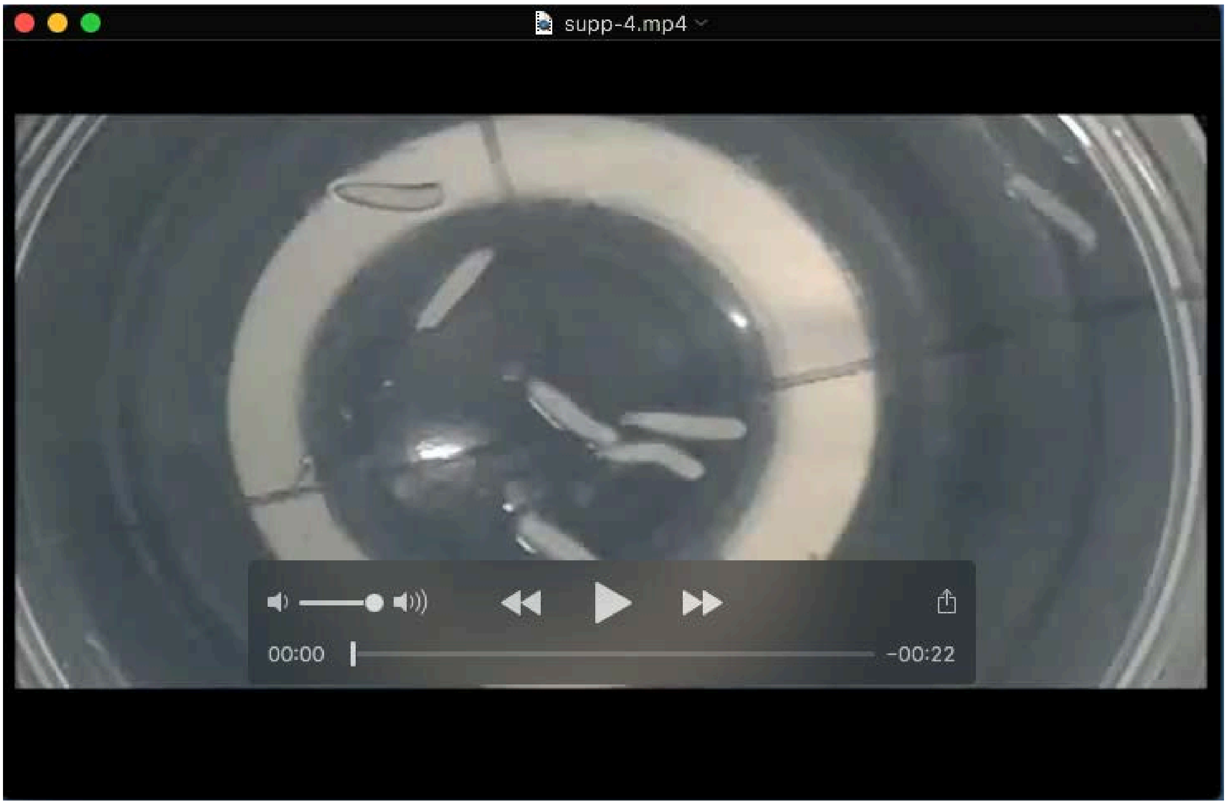

Movie S3. The startle response of *fmr1* mutants to the pulse. It is easier to detect the startle response in *fmr1* mutants than CS.

Biology Open • Supplementary information

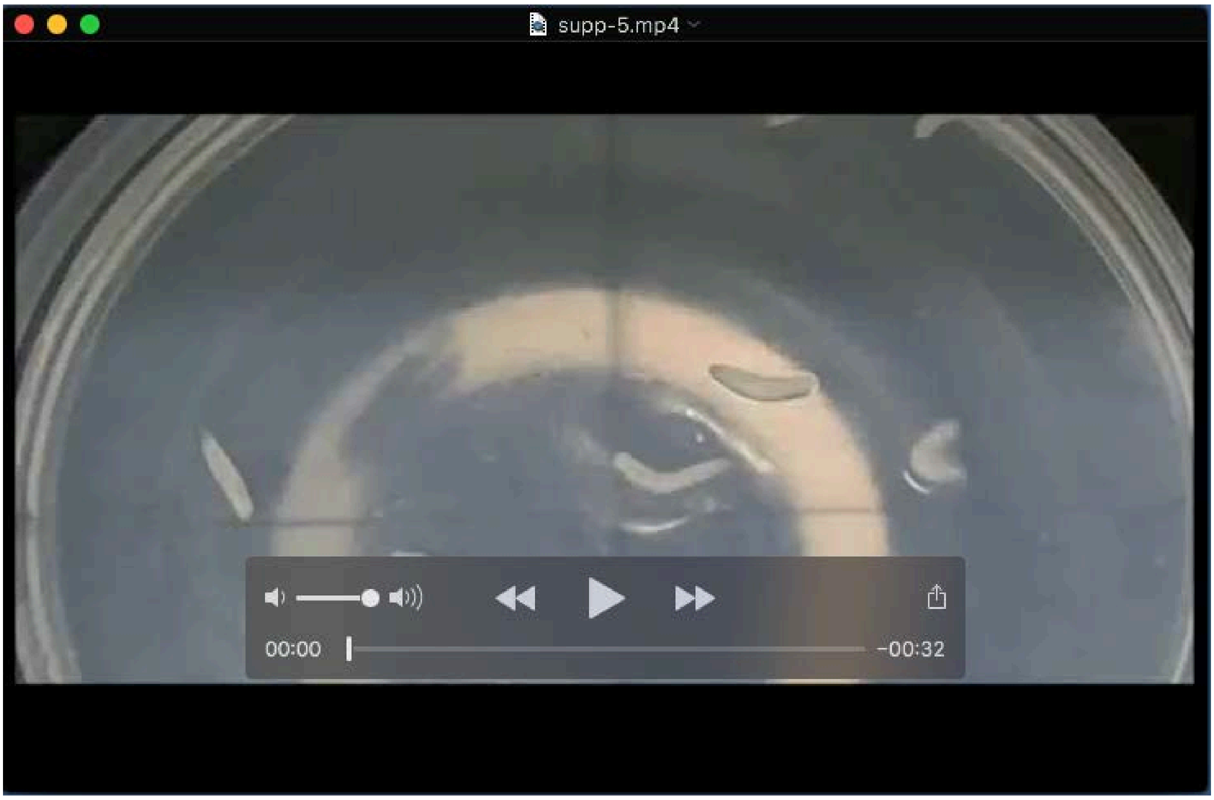

Movie S4. The startle response to the pulse of *fmr1* mutants with the precedent prepulse at intervals of 0.3 s. Even with the prepulse, *fmr1* mutants show strong responses to the pulse, suggesting PPI suppression.
